# Analysis of segregation distortion and its relationship to hybrid barriers in rice

**DOI:** 10.1186/s12284-014-0003-8

**Published:** 2014-08-07

**Authors:** Reflinur, Backki Kim, Sun Mi Jang, Sang-Ho Chu, Yogendra Bordiya, Md Babul Akter, Joohyun Lee, Joong Hyoun Chin, Hee-Jong Koh

**Affiliations:** Department of Plant Science, Plant Genomics and Breeding Institute, and Research Institute for Agriculture and Life Sciences, Seoul National University, Seoul, 151-921 Korea; Indonesian Center for Agricultural Biotechnology and Genetic Resources Research and Development, IAARD, Bogor, Indonesia; Department of Applied Bioscience, Konkuk University, Seoul, 143-701 Korea; International Rice Research Institute, Los Banos, Philippines

**Keywords:** Segregation distortion, Linkage map, Reproductive barrier

## Abstract

**Background:**

Segregation distortion (SD) is a frequently observed occurrence in mapping populations generated from crosses involving divergent genotypes. In the present study, ten genetic linkage maps constructed from reciprocal F_2_ and BC_1_F_1_ mapping populations derived from the parents Dasanbyeo (*indica*) and Ilpumbyeo (*japonica*) were used to identify the distribution, effect, and magnitude of the genetic factors underlying the mechanisms of SD between the two subspecies.

**Results:**

SD loci detected in the present study were affected by male function, female function, and zygotic selection. The most pronounced SD loci were mapped to chromosome 3 (transmitted through male gametes), chromosome 5 (transmitted through male gametes), and chromosome 6 (transmitted through female gametes). The level of SD in BC_1_F_1_ populations which defined by chi-square value independence multiple tests was relatively low in comparison to F_2_ populations. Dasanbyeo alleles were transmitted at a higher frequency in both F_2_ and BC_1_F_1_ populations, suggesting that *indic* a alleles are strongly favored in inter-subspecific crosses in rice. SD loci in the present study corresponded to previously reported loci for reproductive barriers. In addition, new SD loci were detected on chromosomes 2 and 12.

**Conclusion:**

The identification of the distribution of SD and the effect of genetic factors causing SD in genetic mapping populations provides an opportunity to survey the whole genome for new SD loci and their relationships to reproductive barriers. This provides a basis for future research on the elucidation of the genetic mechanisms underlying SD in rice, and will be useful in molecular breeding programs.

**Electronic supplementary material:**

The online version of this article (doi:10.1186/s12284-014-0003-8) contains supplementary material, which is available to authorized users.

## Background

Segregation distortion (SD) is defined as a deviation of the observed genotypic frequency from expected Mendelian segregation ratios. Genetic elements that cause SD are increasingly being recognized as a powerful evolutionary force (Sandler and Novitski [1957]), and it is thought that SD is due to a number of causes including genetic, physiological, and/or environmental factors (Liu et al. [2008]; Matsushita et al. [2003]; Wang et al. [2009]; Xu et al. [1997]).

Several molecular mechanisms of SD have been reported in plants (Fishman et al. [2008]; Fishman et al. [2001]; Fishman and Willis, [2005]; Harushima et al. [2002]; Koide et al. [2008a]; Koide et al. [2008b]; Koide et al. [2011]). As reproductive isolation is a result of multiple genes acting at various stages throughout the life history of an organism, further dissection of SD regions in different populations and cross combinations is of key importance for breeding programs. In rice, previous studies detected loci linked with SD in both single and multiple crosses (Harushima et al. [2001], [2002]; Wang et al. [2009]). Use of abundant DNA markers allowed development of high-density linkage maps for rice, and these were used to survey the whole genome for SD loci (Causse et al. [1994]; Harushima et al. [1996]; Yano et al. [1998]). Most of the barriers were mapped at different loci and demonstrated to have formed after *japonica*–*indica* differentiation (Harushima et al. [2001], [2002]).

Since varietal differentiation in *O. sativa* is complex, data obtained from a single cross are not representative of the full range of differentiating Asian rice (Harushima et al. [2002]). Thus, Harushima et al. ([2002]) used multiple crosses to investigate the diverse variation of reproductive barriers in rice. In that experiment, 33 reproductive barriers were identified, 15 of which affected the gametophyte and 18 of which altered viability of the zygote. However, as a probable consequence of the cross combinations used, the experiment was unable to clearly distinguish between female and male gametophytic selection-originated SDs. Therefore, the use of different mapping populations would be likely to reveal distorted loci or causes of SD that were not identified previously.

In the present study, two reciprocal F_2_ and eight BC_1_F_1_ populations developed from two Korean popular rice cultivars, Ilpumbyeo (*japonica*) and Dasanbyeo (*indica*), were used to investigate SD. Nuclear genetic or cytoplasmic factors influencing SD rates were inferred from the distortion patterns of DNA markers in reciprocal F_2_ populations. Gametophytic and zygotic selection as mechanisms underlying SD were inferred from distorted marker patterns in BC_1_F_1_ populations conferring female- or male-segregating populations. Information regarding the loci and other factors responsible for SD in rice are important for the selection of breeding cultivars, and could also be beneficial for the development of molecular breeding programs. In this study, we aimed to map the genomic regions showing SD and to characterize whether gametic- or zygotic selection caused SD at each locus.

## Results

### Construction of linkage maps in two F_2_ and eight BC_1_F_1_ populations

Total map length, average distance between adjacent markers, and the linkage map coverage of the genome constructed from reciprocal F_2_ and BC_1_F_1_ populations was variable in the different mapping populations (Table 1). Physical locations of both end markers for each chromosome and linkage map coverage of the genome constructed from these populations in details were presented in Additional file 1: Table S1. The total map length in the Ilpumbyeo/Dasanbyeo (ID) population was 1391.1cM, with an average distance of 13.3cM between adjacent markers. In the reciprocal cross (DI), total map length and average distance were 1462.7cM and 13.9cM, respectively. With a few exceptions, map lengths in female-segregating populations (BC_1_F_1_ populations in which F_1_ was used as a female parent) were generally longer than those in male-segregating population (BC_1_F_1_ populations in which F_1_ was used as a male parent) (Table 1). Reduction in map length was extreme when Ilpumbyeo as a maternal parent was crossed with F_1_ as a pollen parent. The map lengths of ID//I and DI//I were 1424.1cM and 1514.8cM, respectively, while those of I//ID and I//DI were 525.8cM and 787.3cM, respectively (Table 1). This indicates that the frequency of recombination between marker pairs in I//ID and I//DI is lower than those in ID//I and DI//I.

**Table 1:** **Comparison of total map length and average distance between adjacent markers in genetic linkage map between reciprocal F**_**2**_ **and BC**_**1**_ **F**_**1**_ **populations generated from Dasanbyeo and Ilpumbyeo parents**

Population	Generation	Number of tested loci	Mapped loci	Total map length (cM)	Average distance btw markers (cM)	Map coverage(%)^a^	
						Genetic map	Physical map
I/D	F_2_	107	103	1391.1	13.3	91.0	79.63
D/I	F_2_	107	102	1462.7	13.9	96.0	79.63
DI//D	BC_1_F_1_	144	139	1495.6	11.4	80.2	89.75
D//DI	BC_1_F_1_	144	142	1119.2	8.8	76.6	90.95
ID//I	BC_1_F_1_	144	142	1424.1	11.7	75.3	88.11
I//ID	BC_1_F_1_	144	132	525.8	5.6	34.6	90.95
ID//D	BC_1_F_1_	144	140	1434.7	11.2	73.0	89.86
D//ID	BC_1_F_1_	144	139	1043.2	8.2	69.2	86.84
DI//I	BC_1_F_1_	144	140	1514.8	11.7	89.2	92.11
I//DI	BC_1_F_1_	144	135	787.3	6.5	51.4	85.14

^a^map coverage in genetic and physical maps was based on the reference of Nipponbare/Kasalath map (Harushima *et al.*[1998]) and IRGSP build5 (http://www.rgp.dna.affrc.go.jp/), respectively.

ID = F_2_ of Ilpumbyeo x Dasanbyeo; DI = F_2_ of Dasanbyeo x Ilpumbyeo.

ID//D = Ilpumbyeo/Dasanbyeo//Dasanbyeo, ID//I = lpumbyeo/Dasanbyeo//Ilpumbyeo.

DI//D = Dasanbyeo/Ilpumbyeo//Dasanbyeo,DI//I = Dasanbyeo/Ilpumbyeo//Ilpumbyeo.

I//ID = Ilpumbyeo//Ilpumbyeo/Dasanbyeo; I//DI = Ilpumbyeo//Dasanbyeo//Ilpumbyeo.

D//ID = Dasanbyeo//Ilpumbyeo/Dasanbyeo; and D//DI = Dasanbyeo//Dasanbyeo/Ilpumbyeo.

### SD of DNA markers in two reciprocal F_2_ populations

On the whole, considering the single test performed at every marker locus, significant (acceptance level: 0.05) deviation with respect to expected Mendelian ratio in two F_2_ populations was found at a high number of distorted markers on almost all chromosomes (Table 2).

**Table 2:** **Number of regions of segregation distortion loci detected in two reciprocal F**_**2**_ **and eight BC**_**1**_ **F**_**1**_ **populations generated from Ilpumbyeo and Dasanbyeo**

Pop.^a^	Mapped loci	Loci of SD		Number of regions of SD^d^	Chromosomal regions of SD	Favored genotype (ratio, %)					
						Ilpumbyeo (II)		Heterozygote (ID or DI)		Dasanbyeo (DD)	
		Single test^b^	Multiple test^c^			Single test	Multiple test	Single test	Multiple test	Single test	Multiple test
ID	103	45 (43.7%)	27 (26.2%)	10	Chr.1 (1), Chr.3 (2),Chr.4 (1), Chr.5 (1), Chr.6 (1), Chr.9 (1), Chr.12 (3)	-	-	11 (24.4%)	4 (14.8)	34 (75.6%)	23 (85.2%)
DI	102	41 (40.2%)	20 (19.6%)	8	Chr.1 (1), Chr.3 (1), Chr.4 (1), Chr.5 (1), Chr.6 (1), Chr.12 (3)	1 (2.5%)	-	14 (34.1%)	4 (20%)	26 (63.4%)	16 (80%)
ID//D	140	7 (5.0%)	0	0		-	-	3 (42.9%)	-	4 (57.1%)	-
ID//I	142	28 (19.7%)	7 (4.9%)	1	Chr.6 (1)	18 (64.3%)	5 (71.4%)	10 (35.7%)	2 (28.6%)	-	-
DI/D	139	23 (15.8%)	5 (3.6%)	3	Chr.1 (1), Chr.6 (1), Chr.12 (1)	-	-	10 (43.5%	1 (20%)	13 (56.5%)	4 (80%)
DI//I	140	11 (7.9%)	7 (5%)	1	Chr.6 (1)	5 (45.5%)	-	6 (54.5%)	7 (100%)	-	-
I//ID	132	21 (15.9%)	11 (8.3%)	4	Chr.1 (1), Chr.5 (1), Chr.8 (1), Chr.11 (1)	21 (100%)	11 (100%)	-	-	-	-
I//DI	135	25 (18.5%)	2 (2.2%)	1	Chr.12 (1)	1 (4.0%)	2 (100%)	24 (96.0%)	-	-	-
D//ID	139	39 (28.1%)	11 (7.9%)	5	Chr.1 (1), Chr.3 (1), Chr.5 (1), Chr.8 (1), Chr.12 (1)	-	-	2 (5.1%)	-	37 (94.9%)	11 (100%)
D//DI	142	44 (31.0%)	18 (12.7%)	6	Chr.2 (1), Chr.3 (1), Chr.5 (2), Chr.8 (1), Chr.12 (1)	-	-	5 (11.4%)	-	39 (88.6%)	12 (100%)

^a^refer to Table 1 for abbreviations.

^b^Observed segregation to Mendelian expectation was tested using a Chisquare value by single testing (randomly selected single marker).

^c^Observed segregation to Mendelian expectation was tested using a Chisquare value by multiple testing (sequential Bonferroni correction of *P*-value) across loci separately within each cross.

^d^SD is abbreviation of segregation distortion.

Overall, 45 (43.7%) and 41 (40.2%) loci were significantly distorted from the expected Mendelian segregation ratios in the ID and DI populations, respectively (Table 2). With this acceptance level (alpha 0.05), it can be suggested that 5% of the non-Mendelian segregation were caused by chance. However, to detect false H_0_ upon applying the sequential Bonferroni method, the number of possible SD was extremely reduced. After applying Bonferroni method to segregation data, significant results in 27 out of 45 SD loci in ID population were obtained, which means that 26.2% of the total number of segregation analyzed were non-Mendelian. On the other hand, we obtained significant results in 20 out 41 SD loci in DI population, which means that the SD level decreased from 40.2% to 19.6% (Table 2). Most of the distorted markers in both F_2_ populations were skewed towards Dasanbyeo alleles, indicating that *indica* alleles were transmitted at higher frequency than *japonica* alleles.

### Nuclear and cytoplasmic effects on SD in reciprocal F_2_ populations

It can be inferred that nuclear genetic factors are responsible for SD if markers are similarly distorted in both reciprocal F_2_ populations. Conversely, a cytoplasmic effect can be inferred if the markers are distorted in only one of the reciprocal F_2_ populations. A total of 18 markers were distorted in both F_2_ populations (Additional file 2: Table S2), and were thus caused by nuclear genetic factors. Eleven markers deviated from the expected Mendelian segregation ratio in only one of the two reciprocal F_2_ populations (Additional file 2: Table S2), indicating a cytoplasmic effect. Of these 11 markers, 9 showed SD only in the ID population and 2 showed SD only in the DI population. Favored marker genotypes differed between the ID (Ilpumbyeo cytoplasm) and DI populations (Dasanbyeo cytoplasm); specifically, seven of the 9 SD markers (Additional file 2: Table S2) in the ID population favored Dasanbyeo genotypes and the remaining two loci favored heterozygous genotypes, while two SD loci in the DI population favored Dasanbyeo genotypes (Additional file 2: Table S2 and Additional file 3: Figure S1).

### Effect of gametophytic and zygotic factors on SD in reciprocal BC_1_F_1_ populations

Number of regions of SD and the chromosomal regions associated with SD from eight BC_1_F_1_ populations are presented in Table 2 and Table 3, respectively. To determine whether the SD loci detected in the F_2_ populations originated from female function (embryo-sac effect), male function (pollen effect), or post-fertilization selection among zygotes (hereafter, zygotic selection), genotype ratios were compared in pairs of reciprocal backcross to each parent. The significant SD detected in the BC_1_F_1_ populations only when the F_1_ was used as a female parent is indicative of SD due to the female effect since there was no segregation in male parent. By contrast, SD detected in the BC_1_F_1_ population only when the F_1_ male was used as a parent is indicative of the male effect since there was no segregation in the female parent. However, when markers showed SD in both backcrosses, SD is indicative of zygotic selection. Our data thus suggest three mechanisms that lead to SD, as follows: SD through female function, SD through male function, and SD through zygotic selection.

**Table 3:** **Characterization of segregation distortion loci underlying the transmission of gametes through female, male and zygotic selections**

Chr^a^	Marker	Position	%DD^b^	%DD	%DD	%DD	%DD	%DD	%II	%II	%II	%II	Mechanism^c^
		(Mbp)	(ID)	(DI)	(ID//D)	(D//ID)	(DI//D)	(D//DI)	(ID//I)	(I//ID)	(DI//I)	(I//DI)	
1	S01022	4.38	0.36^**(DD)^	0.34^**(DD)^		0.78^**(DD)^	0.71^**(DD)^						Zygotic selection
1	S01038	7.46	0.34^**(DD)^	0.36^**(DD)^		0.75^**(DD)^	0.73^**(DD)^						Zygotic selection
1	S01157B	39.8								0.83^**(II)^			mSD
1	S01160	40.80								0.84^**(II)^			mSD
1	S01181B	43.20								0.84^**(II)^			mSD
2	S02126	29.91						0.68^**(DD)^					mSD
2	S02135	31.48						0.69^**(DD)^					mSD
3	S03027	5.71	0.38^**(DD)^	0.34^**(DD)^				0.77^**(DD)^					mSD
3	S03041	8.90	0.38^**(DD)^	0.41^**(DD)^		0.79^**(DD)^		0.87^**(DD)^					mSD
3	S03046	10.14	0.41^**(DD)^	0.41^**(DD)^		0.79^**(DD)^		0.91^**(DD)^					mSD
3	S03048	10.75	0.41^**(DD)^	0.40^**(DD)^		0.83^**(DD)^		0.89^**(DD)^					mSD
3	S03065	14.43	-	-		0.83^**(DD)^		0.94^**(DD)^					mSD
3	S03130	29.83	0.32^**(DD)^										Cytoplasm effect
3	S03136	30.11	0.33^**(DD)^										Cytoplasm effect
4	S04113	32.61	0.22^**(H)^	0.21^**(H)^									Nuclear effect
4	S04120	33.60	0.14^**(H)^	0.16^**(H)^									Nuclear effect
5	S05004B	0.29	0.41^**(DD)^	0.33^**(H)^		0.73^**(DD)^		0.78^**(DD)^					mSD
5	S05009	0.84	0.39^**(DD)^	0.26^**(H)^		0.73^**(DD)^		0.83^**(DD)^					mSD
5	S05029	3.42	0.34^**(DD)^	0.35^**(DD)^		0.75^**(DD)^		0.81^**(DD)^					mSD
5	S05030A	3.66	0.33^**(DD)^	0.33^**(DD)^		0.77^**(DD)^		0.80^**(DD)^					mSD
5	S05030B	3.66	0.33^**(DD)^	0.33^**(DD)^		0.77^**(DD)^		0.77^**(DD)^					mSD
5	S05032	4.29		0.34^**(DD)^				0.74^**(DD)^		0.84^**(H)^			mSD + Cytoplasm effect
5	S05036	4.71		0.35^**(DD)^				0.74^**(DD)^		0.80^**(H)^			mSD + Cytoplasm effect
5	S05045	6.97	-	-				0.73^**(DD)^		0.80^**(H)^			mSD
5	S05064	16.99						0.73^**(DD)^		0.84^**(H)^			mSD
5	S05077A	20.10						0.72^**(DD)^		0.80^**(H)^			mSD
6	S06018	4.74	0.36^**(DD)^	0.37^**(DD)^			0.68^**(DD)^		0.34^**(H)^		0.26^**(H)^		fSD
6	S06031	5.68	0.36^**(DD)^	0.36^**(DD)^			0.67^**(DD)^		0.30^**(H)^		0.25^**(H)^		fSD
6	S06040	7.83							0.26^**(H)^		0.19^**(H)^		fSD
6	S06053	8.83							0.22^**(H)^		0.22^**(H)^		fSD
6	S06065A	14.53							0.29^**(H)^		0.23^**(H)^		fSD
8	S08060	17.27				0.25^**(H)^		0.33^**(H)^		0.84^**(II)^			mSD
8	S08066	18.91				0.23^**(H)^		0.31^**(H)^		0.80^**(II)^			mSD
8	S080075	20.65				0.23^**(H)^		0.30^**(H)^		0.88^**(II)^			mSD
8	S08080B	21.33				0.23^**(H)^		0.27^**(H)^		0.84^**(II)^			mSD
8	S08090	23.08				0.19^**(H)^		0.28^**(H)^		0.84^**(II)^			mSD
9	S09065	17.91	0.19^**(H)^										Cytoplasm effect
9	S09075A	19.58	0.20^**(H)^										Cytoplasm effect
11	S11004A	1.08								0.88^**(II)^			mSD
11	S11006	1.27								0.88^**(II)^			mSD
12	S12005	0.33	0.90^**(DD)^	0.89^**(DD)^									Nuclear effect
12	S12009A	0.63	0.39^**(DD)^	0.43^**(DD)^		0.78^**(DD)^		0.71^**(DD)^				0.18^**(H)^	mSD
12	S12011B	1.88	0.40^**(DD)^	0.38^**(DD)^		0.75^**(DD)^		0.69^**(DD)^				0.16^**(H)^	mSD
12	S12030	3.84	0.36^**(DD)^										Cytoplasm effect
12	S12039B	5.57	0.31^**(DD)^										Cytoplasm effect
12	S12055B	15.57	0.35^**(DD)^										Cytoplasm effect
12	S12066	19.44	0.33^**(DD)^				0.23^**(H)^						fSD + Cytoplasm effect
12	S12071	19.66	-	-			0.24^**(H)^						fSD
12	S12091	23.65	-	-			0.24^**(H)^						fSD
12	S12097B	25.00	0.41^**(DD)^				0.25^**(H)^						fSD + Cytoplasm effect

^a^indicates chromosomal regions showing segregation distortion (SD) at least in one population.

^b^genotypic ratios were tested against the expected Mendelian expectation to determine significant of SD (χ^2^ with 2 df for F_2_, 1 df for backcrosses: **indicating locus showed significant deviation from Mendelian segregation ratio (Bonferroni corrected). DD and II are abbreviation for genotype frequency (%) of Dasanbyeo and Ilpumbyeo, respectively. The direction of skewness is followed in bracket where DD for Dasanbyeo, II for Ilpumbyeo homozygous, and H for heterozygous genotypes. (-) indicating the primers were not tested in certain populations.

^c^factor causing segregation distortion, such as male function (mSD), female function (fSD), or zygotic selection. Male function (mSD) is defined if segregation ratios of those markers are significantly distorted from the expected mendelian segregation ratios in BC_1_F_1_ types conferring male-segregating population, but in its reciprocal cross was normally segregated according to mendelian pattern. Whereas, female function (fSD) causing SD is defined by the markers that only distorted in the type of BC_1_F_1_ conferring female-segregating population and normally segregated in its reciprocal cross. On the other hand, zygotic selection causing SD is explained by those markers which are distorted in BC_1_F_1_ types conferring both female-and male segregating populations.

### SD through female function

On the basis of SD patterns observed in BC_1_F_1_ conferring female-segregating populations (F_1_ plants were used as female parents), two chromosomal regions (chromosomes 6 and 12) harbored SD loci putatively influenced by female function, hereafter termed female-specific SD loci or fSD (Table 3). Of these, female-function influence on SD was most pronounced in chromosome 6, as indicated by segregation distortion of the marker loci in three of the female-segregating populations (ID//I, DI//D, DI//I), but not in the reciprocal backcrosses (I//ID, D//DI, I//DI). Thus, a region encompassing the markers S06018 and S06031 on chromosome 6 was subject to significant transmission bias in all female-segregating populations with the exception of ID//D (Table 3). Moreover, in the ID//I and DI//I populations, the marker distortion affect extended to the neighboring region (S06040B, S06053, and S06065), resulting in the presence of severely distorted markers across a large portion of chromosome 6 (Additional file 4: Figure S2). In contrast, inheritance of these markers was undistorted in the male-segregating population, implying that the SD was caused by female-specific function only and that pollen grain competition is not responsible for SD detected on chromosome 6. Our finding is in accordance with the earlier studies about SD in *japonica-indica* hybrids conducted by Lin et al. ([1992]). In their study, when pollen from F_1_ hybrids were used for backcrossing, no segregation distortion was found for marker gene loci on chromosome 6 and thus, they suggested that SD of markers on chromosome 6 is caused through partial female gamete abortion in *indica-japonica* hybrids. In addition, SD through female function on chromosome 6 could also be caused and explained by reducing of spikelet fertility since the certain SD regions was concurred with *qSF6.1* (S06031-S06040B) and *qSF6.2* (S06040B-S06053) loci responsible for hybrid fertility genes in our previous study (Reflinur et al. [2012]).

Dasanbyeo alleles were over-represented at all distorted markers on chromosome 6 in the three populations, as determined by the skew direction. This indicates that Dasanbyeo embryo sacs were preferred during fertilization by both Dasanbyeo and Ilpumbyeo pollen, thus causing the distortion of certain regions on chromosome 6. The same chromosome 6 markers segregated normally in the ID//D population, suggesting that this might be the best cross combination with which to obtain new recombinants among breeding BC_1_F_1_ populations. The utility of this combination is further supported by the low observed frequency of SD, in which only seven (5%) of the 140 sequence-tagged site STS markers (0% after corrected-Bonferroni) were distorted (Table 2). Although further study is required to determine the factors influencing low SD frequency in ID//D, this backcross direction can nevertheless be used to eliminate hybrid barriers causing SD in *indica-japonica* crosses.

Unlike evidence of SD on chromosome 6 which could be explained by female function on SD through 3 out 4 female-backcrossing populations, fSD on chromosome 12 (S12066, S12071, S12091, and S12097B) was found inconsistent. This region was distorted only in DI//D population while certain region was normally segregated in a Mendelian fashion in other three female-segregating populations (ID//D, ID//I, and DI//I). This inconsistency might be explained by preferential fertilization in which male gametophyte with D genotype interacts with maternal D genotype. The direction of skewness in this population was towards heterozygous (DI) genotypes indicating preferential segregation of Ilpumbyo allele during F_1_ female meiosis giving rise to high probability of fertilization between Ilpumbyeo-embryosac and Dasanbyeo-pollen which caused transmission ratio distortion.

### SD through male function

**Figure 1:**
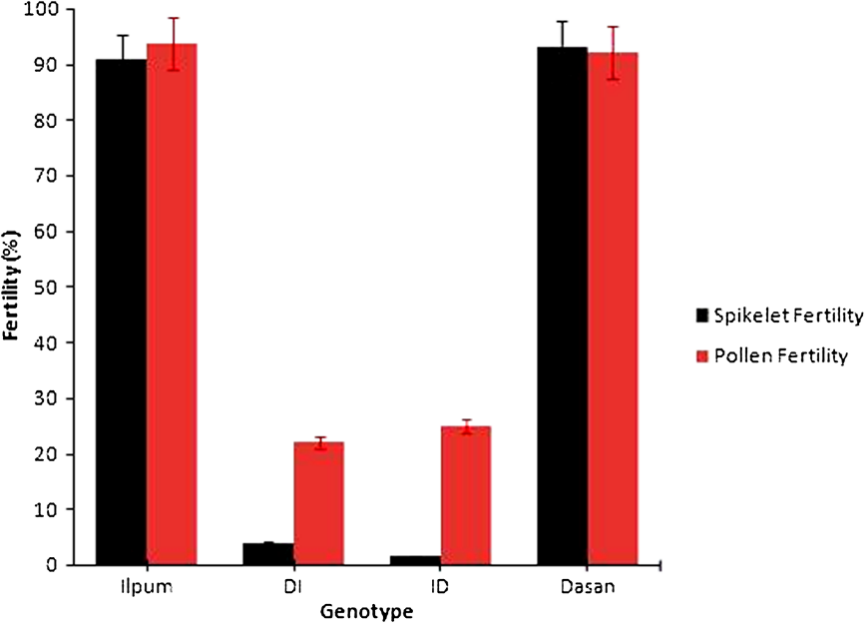
**Pollen and spikelet fertility of parental lines and reciprocal F**_**1**_**plants derived from Ilpumbyeo and Dasanbyeo crosses.** Both reciprocal F_1_ plants showed low fertility of pollen and spikelet.

Seven SD loci putatively influenced by male function, hereafter denoted as male-specific SD loci or mSD, were detected on distorted regions of chromosomes 1, 2, 3, 5, 8, 11 and 12. The regions harboring the most severe distortion patterns were on chromosomes 3, 5 and 8 (Table 3). Markers showing SD on chromosome 3 were distorted in two of the four male-segregating backcross populations, D//ID and D//DI. The skew direction in both D//ID and D//DI was towards Dasanbyeo alleles. Both skew directions imply that Dasanbyeo pollens were preferred to Ilpumbyeo pollens when under competition. Similarly, SD observed on chromosome 5 at the loci S05004B, S05009, S05029, S05030A, and S05030B, were significant in D//ID and D//DI maps but not in their reciprocal backcross populations ID//D and DI//D. The fact that mSD on chromosome 3 and 5 was not observed in either I//ID or I//DI populations reflecting that preferential fertilization might be possible explanation for mSD on certain chromosome. In this case, male gametophytes with D genotype interacted with maternal D genotype loci and preferentially fertilized. Theory suggests that the meiotic drive elements such as gametophytic competition results in preferential fertilization or abortion of gamete or zygote, are the main influence factors of SD in plants (Lyttle, [1991]; Taylor and Ingvarsson [2003]), thus the mechanism showing by certain loci is most likely as a consequence of pollen competition (Table 3). However, the pollen fertility of BC_1_F_1_ progeny was not observed in present study because poor number of backcross population size as a result of reduced fertility in F_1_ pollen and spikelet fertility (Figure 1). In addition, significantly reduced pollen fertility observed in the two reciprocal F_1_ plants was not affected by crossing direction (Figure 1) indicating that there might be no cytoplasmic effect on the expression of pollen fertility. However, it is likely that detected SD loci were explained by the fertilities. In particular D//DI population, the SD region extended further, spreading almost to the distal end of chromosome 5 as also observed in I//ID map (S05032, S05036, S05045, S05064, and S05077A) indicating the severity of the pollen competition effect (Additional file 4: Figure S2). However, the role of pollen competition effect on SD should be further clarified through in depth investigation of pollen fertility in BC_1_F_1_ progeny. As in chromosome 3, distorted regions on chromosome 5 showed a preference for Dasanbyeo alleles. Dasanbyeo was over-represented in D//ID and D//DI, whereas heterozygous was over-represented in I//ID, indicating in both cases that Dasanbyeo pollens were preferred over Ilpumbyeo pollens when under competition.

Male-function influence on SD in chromosomes 8 and 12 was indicated by segregation distortion of the marker loci in three of the male-segregating populations (D//ID, D//DI, I//ID), but not in the reciprocal backcrosses (ID//I, DI//D, ID//I). Thus, a region encompassing the markers S08060 to S08090 on chromosome 8 and that of the markers S12009A and S12011 on chromosome 12 was subject to significant transmission bias in all male-segregating populations with the exception of I//ID (Table 3). However, unlike the case of mSD on chromosome 3, 5 and 12, Ilpumbyeo alleles were over-represented at all distorted markers on chromosome 8 in the three populations, as determined by the skew direction. Several inconsistencies of mSD loci were found on chromosome 1 and 11 which were only distorted in one out of four male-segregating populations. These regions were detected in I//ID population and the effect of distortion at these loci was skewed towards Ilpumbyeo alleles, implying that maternal I preferred male gametophyte with I genotype under pollen competition. Despite the fact that either F_1_ plants developed from ID or DI crosses consist of Ilpumbyeo and Dasanbyeo pollen grains, when the two F_1_ plants were used as male to fertilize the same female parents (Ilpumbyeo) to produce BC_1_F_1_ plants, their effect on distortion was different that indicated by undistorted segregation of these regions in I//DI population. Thus, further study is needed to elucidate molecular mechanism of SD in these regions.

### SD through zygotic selection

In addition to SD occurring as a consequence of female function and male function, zygotic selection was also observed to cause SD in our backcrossing populations. Zygotic selection causing biased transmission would be explained by the significant transmission ratio distortion in both reciprocal backcrosses. Regions exhibiting SD from zygotic selection only were located on the small part of chromosome 1 which indicated by the distortion of S01022 and S01038 markers in the D//ID and DI//D populations (Table 3). Zygotic selection in this region is due to either inviability of indica/japonica hybrid or related to spikelet fertility. Although earlier we were able to get around 100 lines for BC_1_F_1_ whose F_1_ plants were used as female (embryosac source) and male (pollen sources) parents, the viable plants were very poor. Many BC_1_F_1_ plants were died in seedlings stage reflecting hybrid weakness phenomenon in *japonica*/*indica* crosses and the number of lines survived in the field was decreased. Hence, we assumed that zygotic selection in this region is due to abortion of genotype ID or DI reflected by hybrid weakness reducing number of plants survived in the field both in D//ID (52 lines) and DI//D (69 lines). Another possible explanation that zygotic selection affected SD in this region is related to spikelet fertility. Mean value of spikelet fertility of lines showing heterozygous genotypes was lower than those lines carrying homozygous genotypes (data not shown).

## Discussion

### SD across linkage maps

Several gametophytic and zygotic barriers causing deviation of allele frequencies from Mendelian ratios have been reported in inter-specific rice crosses (Harushima et al. [1996]; Harushima et al. [2002]; Koide et al. [2008b]; Wang et al. [2005], [2009]). In the present study, a similar level of SD was identified in two reciprocal F_2_ cross populations, with both nuclear genetic and cytoplasmic factors causing severe SD. This concurs with several prior studies that reported that both nuclear factors (Goloenko et al. [2002]; He et al. [2001]; Liu and Qu [2008]; Wang et al. [2009]) and cytoplasmic factors (Faris et al. [1998]; Goloenko et al. [2002]; Manabe et al. [1999]; Wang et al. [2009]) play a key role in SD.

In our two F_2_ populations, nuclear genetic factors from Dasanbyeo (*indica*) had stronger effects on SD than those from Ilpumbyeo (*japonica*) since most of the distorted loci were skewed in favor of the *indica* alleles (Table 2, Additional file 2: Table S2 and Additional file 3: Figure S1). These results are consistent with previous observations that SD in segregated populations generated from *indica*/*japonica* hybrids favored the *indica* parent (Harushima et al. [2002]; Wang et al. [2009]; Xu et al. [1997]). This suggests that *indica* alleles may be expected to predominate in every inter-subspecific *indica*/*japonica* cross in rice even though the chromosomal regions containing the clusters of distorted markers are not the same. Differences in clusters of distorted markers among populations and/or crossing directions (Harushima et al. [2002]; Xu et al. [1997]) should be the results of different mechanism of SD.

SD in F_2_ populations possessing *indica* cytoplasm strongly favored Dasanbyeo (*indica*) alleles, as did F_2_ populations possessing *japonica* cytoplasm (Additional file 2: Table S2). This was somewhat consistent with previous reports that distortion in segregated populations possessing *indica* cytoplasm favors *indica* alleles, while populations possessing *japonica* cytoplasm did not harbor distortion, favoring a specific parental allele (He et al. [2001]; Peng et al. [2006]; Wang et al. [2009]).

Among chromosomal regions showing SD, chromosomes 3, 5, 6 and 12 exhibited the most severely distorted markers in one and/or both F_2_ populations. Moreover, the marker distortion in these chromosomal regions extended across a large portion of the chromosome, and to the whole arm of the chromosome in some cases (Additional file 3: Figure S1). In F_2_ populations, the type of selection (gametic and/or zygotic) responsible for SD can be determined by using maximum-likelihood models proposed by previous studies (Lorieux et al. [1995]; Pham et al. [1990]; Wang et al. [2005]). The assumptions of the proposed models are theoretically simplified for only one or two SD loci and male gametophytic genes involving SD. However, the mechanisms producing the reproductive barriers observed in inter- and intra-specific crosses in rice are complex and variable within different populations (Wu et al. [2010]). Thus, in the current study, the type of selection affecting SD was investigated by genotyping a common set of markers in a set of all possible reciprocal BC_1_F_1_ populations, which contains female-segregating and male-segregating populations. Biologically, SD could result from selection among gametes and/or zygotes, when an allele at a locus diminishes gametic or zygotic fitness, linked loci will deviate from the expected Mendelian segregation ratio (Alheit et al. [2011]). Therefore in our study, the number of markers used in reciprocal BC_1_F_1_ populations should be increased in order to obtain an improved estimate of biological SD. BC_1_F_1_ genotyping is an effective approach for the investigation of SD since this hybridization strategy was able to identify female- and male-function effects and zygotic-selection effects, as well as the effects of cytoplasm and/or cyto-nuclear interaction, on SD.

Our data showed that the level of SD in BC_1_F_1_ populations was generally low relative to that in F_2_ populations, indicating that the cause of SD was simplified into male or female function in each BC_1_F_1_ population. Nevertheless, Dasanbyeo alleles were transmitted in higher frequencies than Ilpumbyeo alleles in both F_2_ and BC_1_F_1_ populations; this concurs with previous studies suggesting that *indica* alleles are strongly favored in inter-specific crosses of rice (Harushima et al. [2002]; Wang et al. [2009]; Xu et al. [1997]). The level of SD among our BC_1_F_1_ populations also varied greatly. SD frequency was higher in male-segregating populations than in female-segregating populations, suggesting that the SD observed in our study is strongly driven by male function or pollen competition rather than female function (Table 2).

The SD loci in F_2_ populations did not always concur with SD loci in BC_1_F_1_ populations; for example, although significant SD was detected at S05064 in BC_1_F_1_ populations, no SD at S05064 was detected in either of the F_2_ populations. The Dasanbyeo allele at S05064 was favored in D/DI and I/DI, but the Ilpumbyeo allele was favored in I/ID. This conflicting preference for parental alleles depending on the male or female gametes might nullify the potential SD in F_2_ populations. Similarly, several SD loci in BC_1_F_1_ populations were normally segregated in F_2_ populations. Conversely, all the SD loci found in F_2_ populations exhibited SD in BC_1_F_1_ populations, suggesting that the cause of SD loci in F_2_ populations can be explained by female and/or male function and/or zygotic selection.

It is reported that the linkage distance between markers varies at some chromosomal regions in different crosses and types of population, but the order of markers remains highly conserved (Antonio et al. [1996]). In our study, in general, a reduction in the length of the genetic map was observed in male-segregating populations relative to female-segregating populations (Table 1). This suggests that there may have been more recombination in female gametes and thus, recombination frequency between marker pairs in male-segregating populations was lower than those in female-segregating populations. However, the population sizes in this study may not be large enough to draw a conclusion regarding the comparison of recombination frequencies between markers.

### Relationships of SD regions to hybrid barriers in rice

In order to determine the relationship between SD loci and hybrid barriers in rice, comparative analysis was conducted based on physical mapping of each SD locus to the Nipponbare Pseudomolecule assembly annotated by IRGSP Build5 (http://www.rgp.dna.affrc.go.jp/). The chromosomal regions of substantial SD loci found in this study were compared to the regions with quantitative trait loci (QTLs) underlying hybrid sterility/fertility and gametophyte genes reported in previous studies on rice. Chromosomal regions with SD loci in this study were mostly related to, or corresponded with, chromosomal regions showing hybrid barriers (gametophyte or sterility gene) regions (Table 4).

**Table 4:** **Comparison of segregation distortion loci with hybrid barriers from previous studies**^a^ Abbreviations are the same as in Table 3

Chr	Selection type^a^	Marker (range)	Position (Mbp)	Cross^b^	Previous studies shared common regions
1	zygotic	S01011 ~ S01038	4.38 ~ 7.46	D//ID, DI//D	*ga-9*(Xu et al. [1997]); *f1* (Wang et al. [1998]); *qSF1* (Reflinur et al. [2012])
1	mSD	S01157B ~ S01181B	39.8 ~ 43.2	I//ID	GB (Harushima et al. [2001])
2	mSD	S02126 ~ S02135	29.91 ~ 31.48	D//DI	
2					
3	mSD	S03027 ~ S03065	5.71 ~ 14.43	D//ID, D//DII	*ga-2* and *ga-3*(Xu et al. [1997]); *qHPS-3*(Chen et al. [2006]); *f3*(Wang et al. [1998]); *S33*(Jing et al. [2007]); *L3b* and *S3b*(He and Xu [2000]; He et al. [1999]); *sf3.1*(Marri et al. [2005]);*qSF3.1 and qSF3.2* (Reflinur et al. [2012]);
5	mSD	S05004B ~ S05030B	0.29 ~ 3.66	D//ID, D//DI	*f5*(Wang et al. [2006] ); *S24*(kubo et al. [2000]); *S31*(Zhao et al. [2007]); *qSF5.2* (Reflinur et al. [2012]);
5	mSD	S05032 ~ S05077A	4.29 ~ 20.10	D//DI, ,I//ID	GB (Harushima et al. [2002]); *qHPS-5*(Chen et al. [2006]);
6	fSD	S06018 ~ S06065A	4.74 ~ 14.53	ID//I, DI//D, DI//I	*ga-1*, *ga-4* and *ga-5*(Kinoshita [1995]) ; *S5*(Chen et al. [2008]); *S6*(Koide et al. [2008a]); *esa-1*(Liu et al. [2001]); *L6*(He and Xu [2000]); *S8*(Wan et al. [1993]); *qSF6.2* (Reflinur et al. [2012]);
8	mSD	S08060-S08090	17.27 ~ 23.08	I//ID	*S27*(Sobrizal and Yoshimura [2001])
12	mSD	S12009A ~ S12011B	0.63 ~ 1.88	D//ID, D//DI, I//DI	*qSF12.2* (Reflinur et al. [2012]); *lwr12.1*(Li et al. [2004])
12	fSD	S12066 ~ S12097B	19.44 ~ 25.00	DI//D	

^a^Abbreviations are the same as in Table 3.

^b^Abbreviations are the same as in Table 2.

Our comparisons revealed interesting regions corresponding to severely distorted loci on chromosomes 3, 5, and 6. The SD chromosomal region spanning 4.74–14.53 Mbp on chromosome 6 that was influenced by female function overlapped with hybrid barriers (Table 4). Hybrid barrier loci overlapping this region were as follows: *cim* and *Cif,* which are responsible for the cross incompatibility reaction in the male and the female, respectively (Matsubara and Khin-Thidar [2003]); *S1,* which is responsible for gamete elimination in *O. rufipogon* (Koide et al. [2008b]); gametophyte genes *ga-1* and *ga-5* (Kinoshita [1995]); *S5,* a locus with a key effect on embryo sac fertility (Chen et al, [2008]; Yang et al. [2012]); *esa1,* which affects embryo sac abortion (Liu et al. [2001]); *S6,* which affects sex-independent SD affecting male and female gametogenesis (Koide et al. [2008a]), *L6,* a locus affecting pollen fertility (He and Xu [2000]); *qSF6.2,* which affects spikelet fertility (Reflinur et al. [2012]); and the *S8* locus for embryo sac sterility (Wan et al. [1993]). These loci suggest that the chromosomal region affecting reproductive barriers, especially those influenced by female factors, is mostly conserved on chromosome 6.

The most severe SD region affected by male function was detected along chromosome 3 (4.3–24.3 Mbp). This chromosomal region overlapped the following loci: the gametophytic genes *ga-2* and *ga-3* (Xu et al. [1997]); *qHPS-3 ,* which is responsible for pollen sterility (Chen et al. [2006]); *f3* (Wang et al. [1998]); *sf3.1* (Marri et al. [2005]); *S33* (Jing et al. [2007]); *qSF3.1* and *qSF3.2,* loci responsible for spikelet sterility (Reflinur et al. [2012]); and the *L3b* and *S3b* loci, which affect both pollen and spikelet fertility (He and Xu [2000]; He et al. [1999]). Relevant with several findings of sterility loci located in this region, it is likely that male function contributes to hybrid barriers which may lead SD on chromosome 3.

The distorted chromosomal region on chromosome 5 (0.29–20.66Mbp) was affected by male function and overlapped with the following loci: *f5* (Wang et al. [2006]) and *S24* (Kubo et al. [2000]), which affect pollen sterility; *S31,* a locus affecting embryo sac sterility in rice (Zhao et al. [2007]); *qSF5*.2, a locus responsible for spikelet fertility (Reflinur et al. [2012]); *qHSPS-5,* which affects hybrid pollen sterility (Chen et al. [2006]); and a gametophyte barrier (Harushima et al. [2002]).

SD-containing chromosomal regions that did not correspond to or overlap hybrid barriers (either gametophyte or sterility genes) regions were detected on chromosome 2 (region for mSD at 29.91–31.48Mbp of Nipponbare Pseudomolecule) and chromosome 12 (region for fSD at region of 19.44–25.00 Mbp). These findings suggest that the key factors on these chromosomal regions might be new gametophyte regions that influence SD.

One zygotic-selection-affected SD regions on chromosomes 1 overlapped with hybrid barrier regions that were identified in previous studies (Table 4). SD loci on chromosome 1 corresponded to gametophyte barrier, *ga-9* (Xu et al. [1997]), *f1* (Wang et al. [1998]), and *qSF1* (Reflinur et al. [2012]).

Our finding that zygotic selection affects SD on chromosomes 1 conflicts with previous research from Harushima et al. ([2002]), which found that SD on these chromosomal regions was caused by gametophyte barrier. This may be due to the different genetic backgrounds of the backcross populations used in the previous study and in our study. In addition, no reciprocal backcrosses were performed in the previous study to confirm the action of zygotic selection on SD.

## Conclusions

Taken together, based on the SD analysis in reciprocal F_2_ populations, we summarized that both nuclear genetic and cytoplasm factors from Dasanbyeo (*indica*) had stronger effects on SD than those from Ilpumbyeo (*japonica*). The gametophytic (female and male gamete function) and zygotic selection as mechanisms underlying SD could be explained by the distorted marker patterns using early generation of reciprocal backcross populations. The identification of SD distribution and the effect of genetic factors causing SD that were identified in the present study will provide not only a basis for future research on the elucidation of the molecular mechanisms underlying SD in rice, but also information beneficial for breeding strategies.

## Methods

### Plant materials

**Figure 2:**
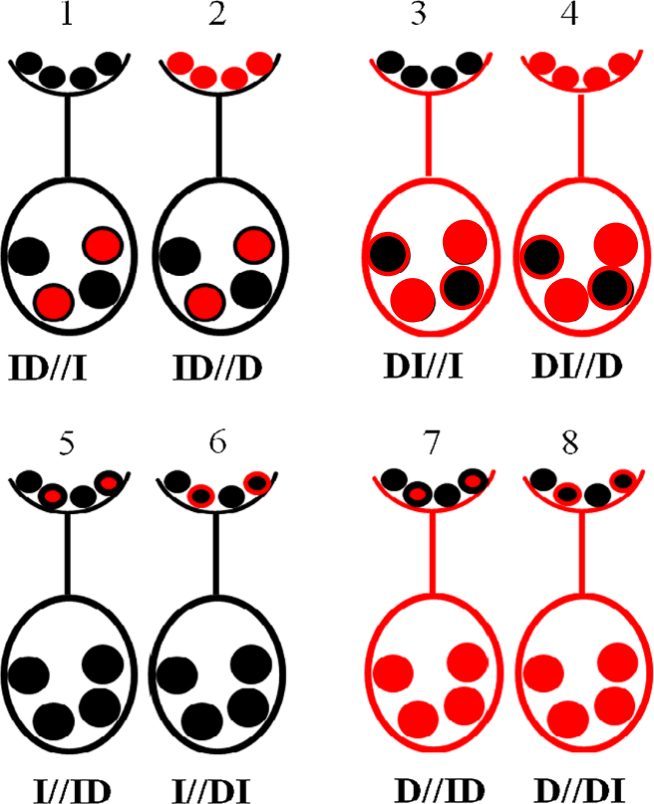
**Backcross population design to distinguish female meiotic drive from male-specific sources of distortion, and to distinguish gametic and zygotic differential selection mechanisms.** Backcross populations were developed from F_1_ plants as female parents (1-4), or as male parents (5-8). Ilpumbyeo (black) and Dasanbyeo (red) genetic background are indicated. Smaller ovals indicate pollen on stigma and larger ovals indicate ovules. Pollen competition occurs only when the F_1_ is the male parent. Loci that exhibit distortion in male-segregating populations (5-8), but no distortion in the female-segregating populations (1-4) thus exhibited male-function- influenced segregation distortion (mSD). The embryo-sac effect on segregation distortion can occur only when the F_1_ is the female parent (1-4). Loci exhibiting deviation from expected Mendelian ratios in female-segregating populations but not in male-segregating populations thus exhibited female-function segregation distortion (fSD). Zygotic selection influencing segregation distortion is indicated by loci with distortion in both backcrosses (F_1_ as female and male parents).

Ten reciprocal crosses consisting of two F_2_ and eight BC_1_F_1_ populations developed from Korean elite *japonica* variety, Ilpumbyeo, and Korean *indica* variety, Dasanbyeo, were used for genetic studies on SD analysis in inter-subspecific crosses of rice. A total of 210 F_2_ progeny (ID) derived from the cross of Ilpumbyeo (female parent) and Dasanbyeo (male parent) and 199 F_2_ progeny (DI) of the reciprocal cross were used. Eight reciprocal BC_1_F_1_ populations were subsequently developed to distinguish female meiotic drive from male-specific sources of distortion, and gametic mechanisms from differential selection against zygote, as described in detail in Figure 2. The numbers of progeny from the BC_1_F_1_ crosses used for mapping and for SD analysis were as follows: 88 from ID//I, 25 from I//ID, 69 from DI//D, 69 from D//DI, 71 from ID//D, 52 from D//ID, 68 from DI//I, and 27 from I//DI population. All plant materials were planted during rice-growing season 2009, at the experimental farm of Seoul National University, Suwon, Korea. Thirty-day-old seedlings were transplanted to irrigated field condition at one seedling per hill with a 30 x 15 cm of spacing between seedlings. Field management was carried out following normal agronomic practices. Fertilizers were properly applied at the rate of 100 kg N ha^−1^, 80 kg P ha^−1^, and 80 kg K ha^−1^ (100-80-80 kg/ha N-P-K).

### Molecular marker analysis

DNA was extracted from young leaf blades according to the method of Causse et al. ([1994]), DNA samples from the F_2_ and BC_1_F_1_ populations were genotyped using 107 and 144 STS primers, respectively. These STS markers are dispersed throughout the rice genome and the primers used were designed by the Crop Molecular Breeding Lab, Seoul National University (Chin et al. [2007]). Polymerase chain reaction (PCR) analysis and visualization of amplicons in the F_2_ and BC_1_F_1_ populations was performed as described previously (Reflinur et al. [2012]).

### Data analysis

Linkage map construction in F_2_ and BC_1_F_1_ populations was performed with Mapmaker/EXP 3.0 program. A LOD threshold of 3.0 was used for declaring linkage (Lander et al. [1987]; Lincoln et al. [1992]), and the Kosambi function was used to convert recombinant value to the genetic distances between the markers (Kosambi [1944]). For the segregation data of each marker, deviations from the Mendelian ratios (1:2:1 ratio for F_2_ populations or 1:1 ratio for BC_1_F_1_ populations) were tested using chi-square analysis. A non-parametric technique using sequential Bonferroni method (Rice [1989]) was applied to the segregation data of each population in order to avoid type-I error deriving from the large number of tests.

## Additional files

## Electronic supplementary material

Additional file 1: Table S1.: Physical locations of both end markers for each chromosome and linkage map coverage of the genome in two reciprocal F_2_ and eight BC_1_F_1_ populations. (XLSX 19 KB)

Additional file 2: Table S2.: Chi-square test for SD of markers and genetic factors influencing distorted markers in two reciprocal F_2_ generated from Ilpumbyeo and Dasanbyeo parents. (DOCX 38 KB)

Additional file 3: Figure S1.: Genotype frequencies of STS markers along chromosomes 3, 5, 6, and 12 in two reciprocal F_2_ populations generated from Ilpumbyeo and Dasanbyeo parents. (DOCX 254 KB)

Additional file 4: Figure S2.: Chromosomal location of pronounced SD loci observed in genetic linkage maps in the two reciprocal F_2_ and eight BC_1_F_1_ populations. (PPTX 194 KB)

Below are the links to the authors’ original submitted files for images.

Authors’ original file for figure 1

Authors’ original file for figure 2
