# Supplementary material for: Analysis of segregation distortion and its relationship to hybrid barriers in rice
Source: Rice (N Y). 2014 Aug 7;7:3. doi: 10.1186/s12284-014-0003-8 (PMC4884001; doi:10.1186/s12284-014-0003-8)
Supplement: Supplementary file 2 — Additional file 2: Table S2.: Chi-square test for SD of markers and genetic factors influencing distorted markers in two reciprocal F2 generated from Ilpumbyeo and Dasanbyeo parents. (DOCX 38 KB) [file 12284_2014_3_MOESM2_ESM.docx]

| **Marker** | **Position^a^** | **Dist (Mbp)** | **(Ilpum/Dasan) F2** | | | **χ2** | **Direction of skewed** | **(Dasan/Ilpum) F2** | | | **χ2** | **Direction of skewed** | **Influenced factor** |
| --- | --- | --- | --- | --- | --- | --- | --- | --- | --- | --- | --- | --- | --- |
|  |  |  | **Genotype** | | |  |  | **Genotype** | | |  |  |  |
|  |  |  | **I/I** | **I/D** | **D/D** |  |  | **D/D** | **D/I** | **I/I** |  |  |  |
| S01022 | Chr1:4384676..4384975 | 4.38 | 33 | 101 | 76 | 17.91^b^ | Dasan | 71 | 101 | 27 | 19.50^b^ | Dasan | Nuclear |
| S01038 | Chr1:7459688..7459931 | 7.46 | 27 | 112 | 71 | 18.48^b^ | Dasan | 71 | 105 | 23 | 22.76^b^ | Dasan | Nuclear |
| S03027 | Chr3:5713283..5713531 | 5.71 | 27 | 104 | 79 | 25.77^b^ | Dasan | 60 | 94 | 45 | 26.35^b^ | Dasan | Nuclear |
| S03041 | Chr3:8900852..8901024 | 8.90 | 14 | 112 | 84 | 47.6 ^b^ | Dasan | 63 | 91 | 45 | 30.05^b^ | Dasan | Nuclear |
| S03046 | Chr3:10137125..10137381 | 10.14 | 14 | 110 | 86 | 49.85^b^ | Dasan | 68 | 88 | 43 | 42.59^b^ | Dasan | Nuclear |
| S03048 | Chr3:10754658..10754836 | 10.76 | 13 | 111 | 86 | 51.44^b^ | Dasan | 75 | 89 | 35 | 42.87^b^ | Dasan | Nuclear |
| S03130 | Chr3:29829040..29829274 | 29.83 | 29 | 113 | 68 | 15.70^b^ | Dasan | 60 | 106 | 33 | 8.18^ns^ |  | Cytoplasm |
| S03136 | Chr3:30109023..30109222 | 30.11 | 28 | 113 | 69 | 16.38^b^ | Dasan | 51 | 96 | 52 | 8.58^ns^ |  | Cytoplasm |
| S04113 | Chr4:32613317..32613471 | 32.61 | 10 | 152 | 47 | 54.67^b^ | Hetero | 42 | 143 | 13 | 46.05^b^ | Hetero | Nuclear |
| S04120 | Chr4:33602147..33602302 | 33.60 | 37 | 144 | 29 | 28.48^b^ | Hetero | 31 | 145 | 23 | 40.9^b^ | Hetero | Nuclear |
| S05004B | Chr5:544779..544987 | 0.54 | 25 | 100 | 85 | 33.58^b^ | Dasan | 65 | 119 | 15 | 31.58^b^ | Hetero | Nuclear |
| S05009 | Chr5:814272..814464 | 0.81 | 24 | 105 | 81 | 29.87^b^ | Dasan | 52 | 136 | 11 | 42.5^b^ | Hetero | Nuclear |
| S05029 | Chr5:3423587..3423788 | 3.42 | 26 | 112 | 72 | 20.15^b^ | Dasan | 70 | 103 | 26 | 18.8^b^ | Dasan | Nuclear |
| S05030A | Chr5:3663509..3663746 | 3.66 | 26 | 115 | 69 | 18.61^b^ | Dasan | 65 | 108 | 26 | 15.88^b^ | Dasan | Nuclear |
| S05030B | Chr5:3663514..3663735 | 3.66 | 28 | 112 | 70 | 16.88^b^ | Dasan | 65 | 109 | 25 | 17.01^b^ | Dasan | Nuclear |
| S05032 | Chr5:4292348..4292594 | 4.29 | 34 | 109 | 65 | 9.03^ns^ |  | 67 | 109 | 23 | 21.27^b^ | Dasan | Cytoplasm |
| S05036 | Chr5:4713483..4713682 | 4.71 | 34 | 109 | 66 | 9.51^ns^ |  | 70 | 105 | 24 | 21.87^b^ | Dasan | Cytoplasm |
| S06018 | Chr6:4737461..4737671 | 4.74 | 32 | 103 | 75 | 17.69^b^ | Dasan | 73 | 100 | 26 | 22.21^b^ | Dasan | Nuclear |
| S06031 | Chr6:5681709..5681920 | 5.68 | 34 | 101 | 75 | 16.31^b^ | Dasan | 71 | 98 | 30 | 16.94^b^ | Dasan | Nuclear |
| S09065 | Chr9:17914403..17914639 | 17.91 | 35 | 134 | 41 | 16.36^b^ | Hetero | 47 | 109 | 43 | 1.70^ns^ |  | Cytoplasm |
| S09075A | Chr9:19575874..19576047 | 19.58 | 33 | 135 | 42 | 17.07^b^ | Hetero | 39 | 114 | 46 | 4.29^ns^ |  | Cytoplasm |
| S12005 | Chr12:331026..331269 | 0.33 | 22 |  | 188 | 23.63^b^ | Dasan | 178 |  | 21 | 21.39^b^ | Dasan | Nuclear |
| S12009A | Chr12:630610..630774 | 0.63 | 16 | 112 | 82 | 41.11^b^ | Dasan | 85 | 101 | 13 | 50.70^b^ | Dasan | Nuclear |
| S12011B | Chr12:1884649..1884804 | 1.89 | 17 | 109 | 84 | 41.75^b^ | Dasan | 74 | 102 | 22 | 26.30^b^ | Dasan | Nuclear |
| S12030 | Chr12:3843516..3843732 | 3.84 | 27 | 107 | 76 | 22.00^b^ | Dasan | 56 | 113 | 30 | 10.46^ns^ |  | Cytoplasm |
| S12039B | Chr12:5567483..5567705 | 5.57 | 36 | 108 | 66 | 20.50^b^ | Dasan | 57 | 108 | 34 | 6.77^ns^ |  | Cytoplasm |
| S12055B | Chr12:15257397..15257562 | 15.57 | 31 | 105 | 74 | 17.61^b^ | Dasan | 52 | 112 | 35 | 6.05^ns^ |  | Cytoplasm |
| S12066 | Chr12:19437623..19437837 | 19.44 | 26 | 114 | 70 | 19.61^b^ | Dasan | 54 | 110 | 35 | 5.84^ns^ |  | Cytoplasm |
| S12097B | Chr12:25000312..25000546 | 25.00 | 20 | 103 | 87 | 42.83^b^ | Dasan | 54 | 111 | 34 | 6.68^ns^ |  | Cytoplasm |

**Table S2 Chi-square test for segregation distortion of markers and genetic factors influencing distorted markers in two reciprocal F_2_ generated from Ilpumbyeo and Dasanbyeo.**

^a^ indicated the physical location of both end markers

^b^ Markers showed significant deviation from Mendelian segregation ratio (Bonferroni-corrected) between observed and expected genotype frequencies according to χ^2^ goodness of fit tests. While markers segregated normally were indicated by ns.
