# Supplementary material for: Analysis of segregation distortion and its relationship to hybrid barriers in rice
Source: Rice (N Y). 2014 Aug 7;7:3. doi: 10.1186/s12284-014-0003-8 (PMC4884001; doi:10.1186/s12284-014-0003-8)
Supplement: Supplementary file 3 — Additional file 3: Figure S1.: Genotype frequencies of STS markers along chromosomes 3, 5, 6, and 12 in two reciprocal F2 populations generated from Ilpumbyeo and Dasanbyeo parents. (DOCX 254 KB) [file 12284_2014_3_MOESM3_ESM.docx]

**
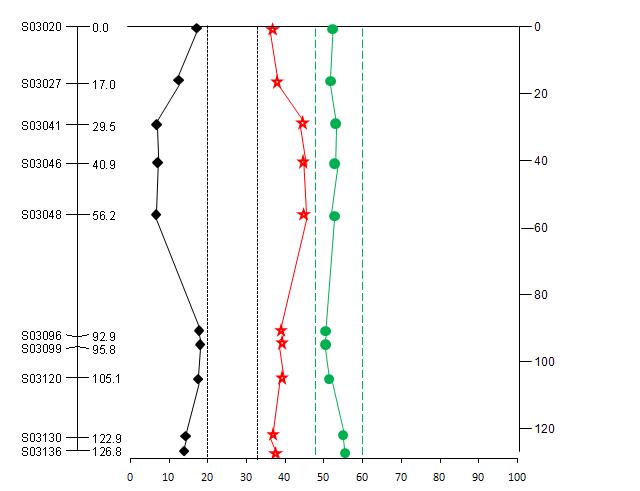

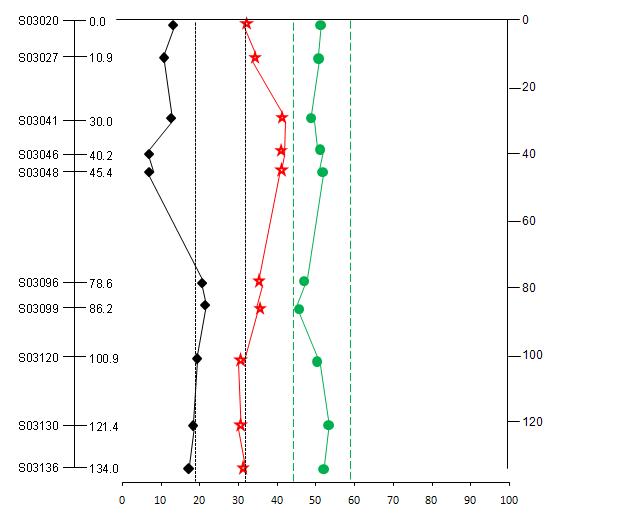
**

A

DI population

ID population


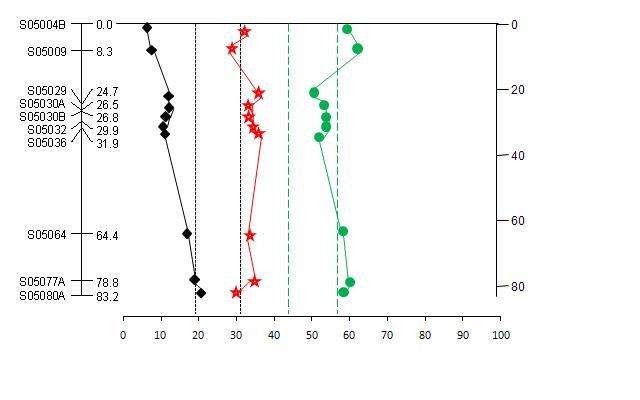

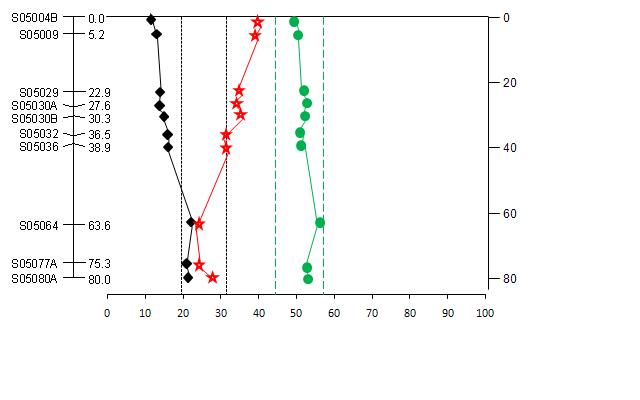


B

DI population

ID population


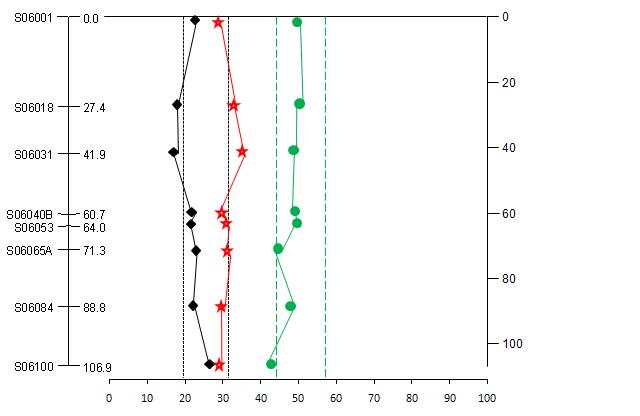

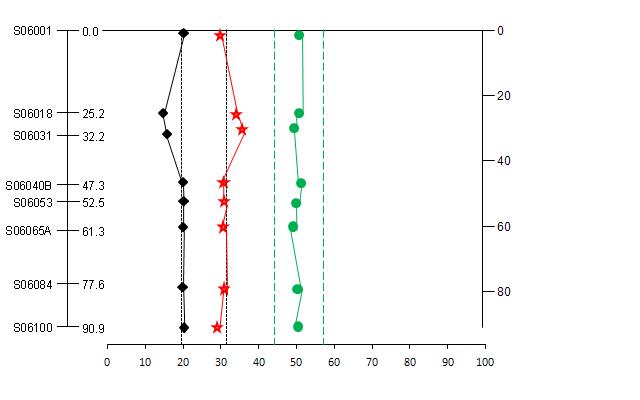


ID population

C

DI population


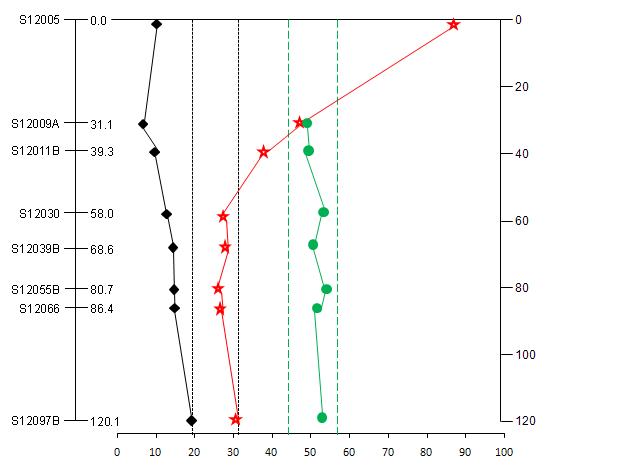

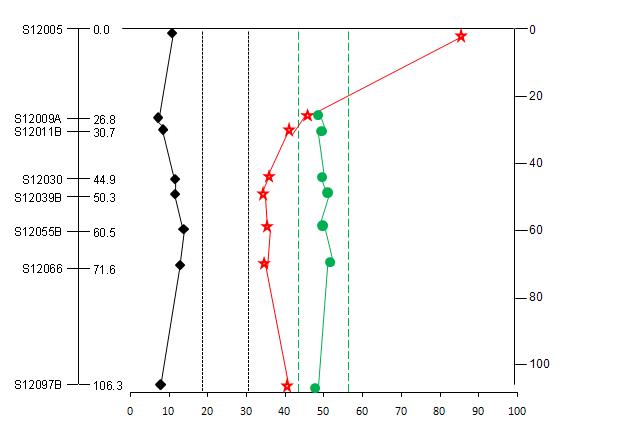


DI population

ID population

D


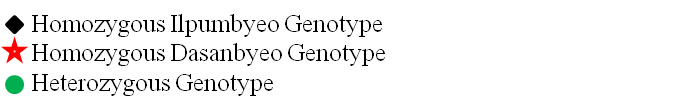


Figure S1. Genotype frequencies of STS markers along chromosome 3 (a), 5 (b), 6 (c), and 12 (d) in two reciprocal F_2_ populations generated from the parents, Ilpumbyeo and Dasanbyeo. The x-axis corresponds to the genotype percentages observed for each marker and the y-axis indicates the genetic linkage map. In normal segregation, the heterozygous and homozygous genotypes account for 50 and 25%, respectively. The 0.95 confidence limits are 43%<y<57% indicated by round dotted lines and 19%<y<31% indicated by square dotted lines for heterozygous and homozygous genotypes, respectively.
