# Supplementary material for: Analysis of segregation distortion and its relationship to hybrid barriers in rice
Source: Rice (N Y). 2014 Aug 7;7:3. doi: 10.1186/s12284-014-0003-8 (PMC4884001; doi:10.1186/s12284-014-0003-8)
Supplement: Supplementary file 4 — Additional file 4: Figure S2.: Chromosomal location of pronounced SD loci observed in genetic linkage maps in the two reciprocal F2 and eight BC1F1 populations. (PPTX 194 KB) [file 12284_2014_3_MOESM4_ESM.pptx]

## Slide 1
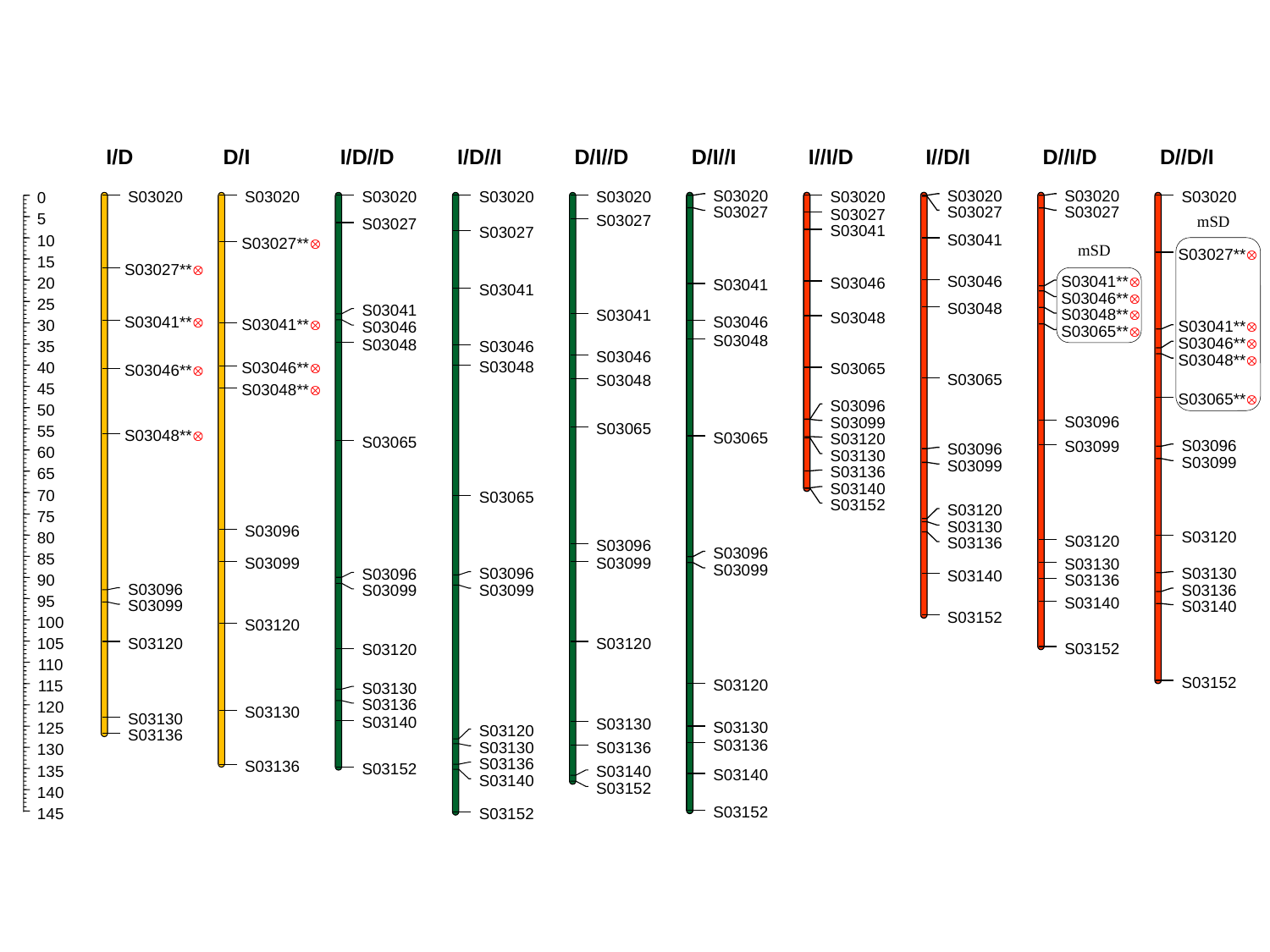

I/D
S03020
S03027**
S03041**
S03046**
S03048**
S03096
S03099
S03120
S03130
S03136
D/I
S03020
S03027**
S03041**
S03046**
S03048**
S03096
S03099
S03120
S03130
S03136
I/D//D
S03020
S03027
S03041
S03046
S03048
S03065
S03096
S03099
S03120
S03130
S03136
S03140
S03152
I/D//I
S03020
S03027
S03041
S03046
S03048
S03065
S03096
S03099
S03120
S03130
S03136
S03140
S03152
D/I//D
S03020
S03027
S03041
S03046
S03048
S03065
S03096
S03099
S03120
S03130
S03136
S03140
S03152
D/I//I
S03020
S03027
S03041
S03046
S03048
S03065
S03096
S03099
S03120
S03130
S03136
S03140
S03152
I//I/D
S03020
S03027
S03041
S03046
S03048
S03065
S03096
S03099
S03120
S03130
S03136
S03140
S03152
I//D/I
S03020
S03027
S03041
S03046
S03048
S03065
S03096
S03099
S03120
S03130
S03136
S03140
S03152
D//I/D
S03020
S03027
S03041**
S03046**
S03048**
S03065**
S03096
S03099
S03120
S03130
S03136
S03140
S03152
D//D/I
S03020
S03027**
S03041**
S03046**
S03048**
S03065**
S03096
S03099
S03120
S03130
S03136
S03140
S03152
0
5
10
15
20
25
30
35
40
45
50
55
60
65
70
75
80
85
90
95
100
105
110
115
120
125
130
135
140
145
mSD
mSD

## Slide 2
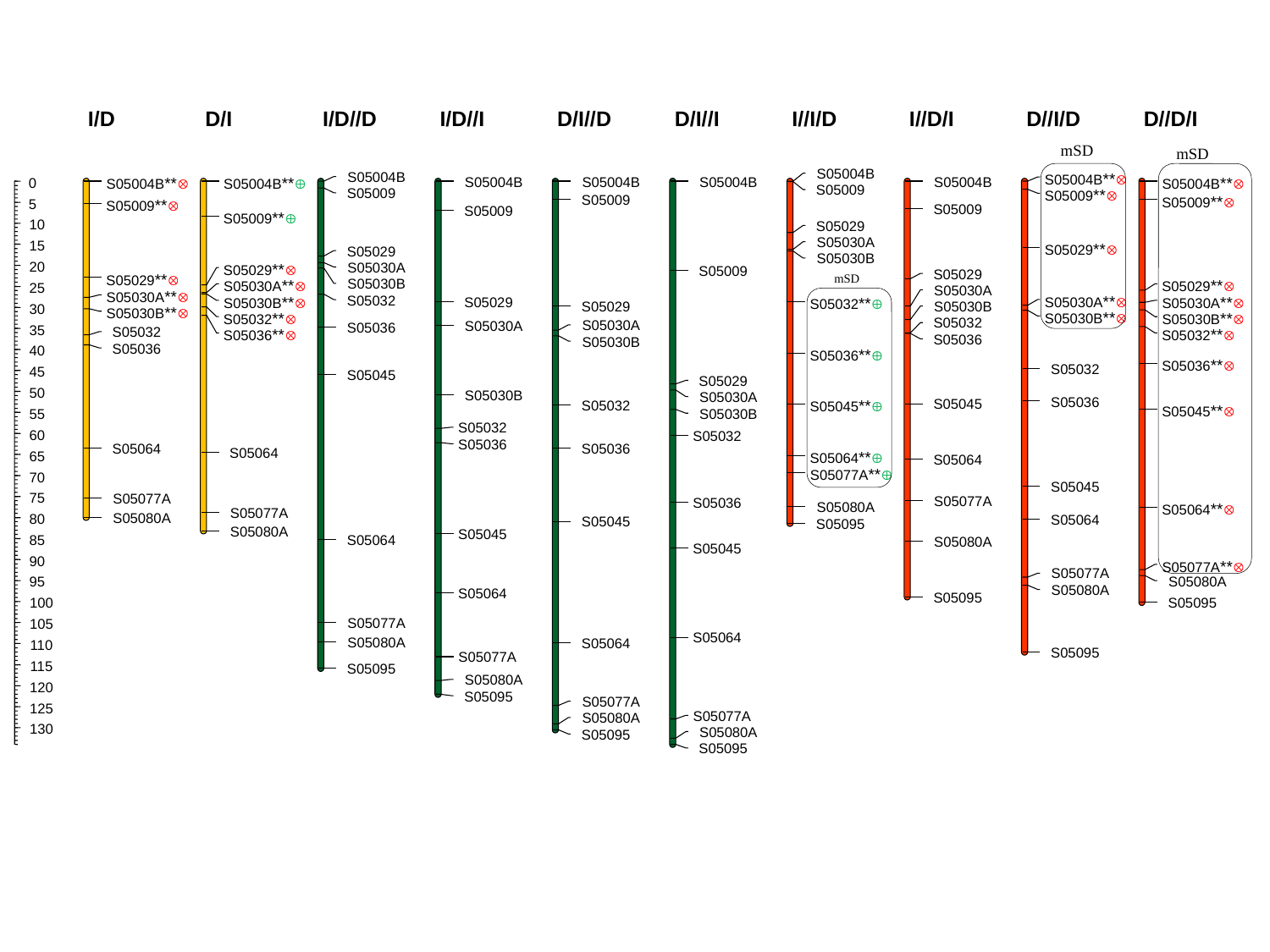

I/D
S05004B**
S05009**
S05029**
S05030A**
S05030B**
S05032
S05036
S05064
S05077A
S05080A
D/I
S05004B**
S05009**
S05029**
S05030A**
S05030B**
S05032**
S05036**
S05064
S05077A
S05080A
I/D//D
S05004B
S05009
S05029
S05030A
S05030B
S05032
S05036
S05045
S05064
S05077A
S05080A
S05095
I/D//I
S05004B
S05009
S05029
S05030A
S05030B
S05032
S05036
S05045
S05064
S05077A
S05080A
S05095
D/I//D
S05004B
S05009
S05029
S05030A
S05030B
S05032
S05036
S05045
S05064
S05077A
S05080A
S05095
D/I//I
S05004B
S05009
S05029
S05030A
S05030B
S05032
S05036
S05045
S05064
S05077A
S05080A
S05095
I//I/D
S05004B
S05009
S05029
S05030A
S05030B
S05032**
S05036**
S05045**
S05064**
S05077A**
S05080A
S05095
I//D/I
S05004B
S05009
S05029
S05030A
S05030B
S05032
S05036
S05045
S05064
S05077A
S05080A
S05095
D//I/D
S05004B**
S05009**
S05029**
S05030A**
S05030B**
S05032
S05036
S05045
S05064
S05077A
S05080A
S05095
D//D/I
S05004B**
S05009**
S05029**
S05030A**
S05030B**
S05032**
S05036**
S05045**
S05064**
S05077A**
S05080A
S05095
0
5
10
15
20
25
30
35
40
45
50
55
60
65
70
75
80
85
90
95
100
105
110
115
120
125
130
mSD
mSD
mSD

## Slide 3
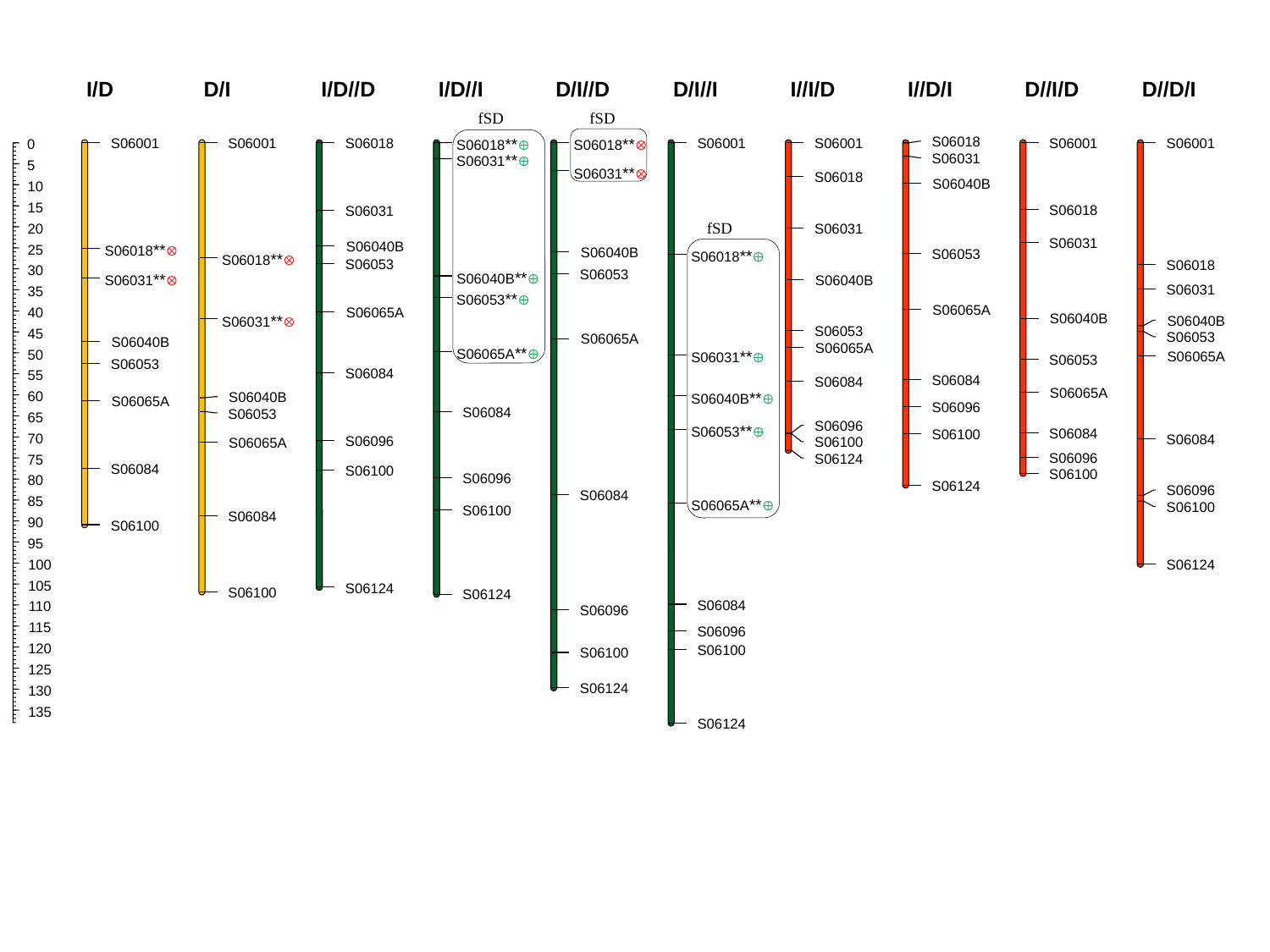

I/D
S06001
S06018**
S06031**
S06040B
S06053
S06065A
S06084
S06100
D/I
S06001
S06018**
S06031**
S06040B
S06053
S06065A
S06084
S06100
I/D//D
S06018
S06031
S06040B
S06053
S06065A
S06084
S06096
S06100
S06124
I/D//I
S06018**
S06031**
S06040B**
S06053**
S06065A**
S06084
S06096
S06100
S06124
D/I//D
S06018**
S06031**
S06040B
S06053
S06065A
S06084
S06096
S06100
S06124
D/I//I
S06001
S06018**
S06031**
S06040B**
S06053**
S06065A**
S06084
S06096
S06100
S06124
I//I/D
S06001
S06018
S06031
S06040B
S06053
S06065A
S06084
S06096
S06100
S06124
I//D/I
S06018
S06031
S06040B
S06053
S06065A
S06084
S06096
S06100
S06124
D//I/D
S06001
S06018
S06031
S06040B
S06053
S06065A
S06084
S06096
S06100
D//D/I
S06001
S06018
S06031
S06040B
S06053
S06065A
S06084
S06096
S06100
S06124
0
5
10
15
20
25
30
35
40
45
50
55
60
65
70
75
80
85
90
95
100
105
110
115
120
125
130
135
fSD
fSD
fSD
