# Supplementary figures and images for: Analysis of segregation distortion and its relationship to hybrid barriers in rice
Source: Rice (N Y). 2014 Aug 7;7:3. doi: 10.1186/s12284-014-0003-8 (PMC4884001; doi:10.1186/s12284-014-0003-8)

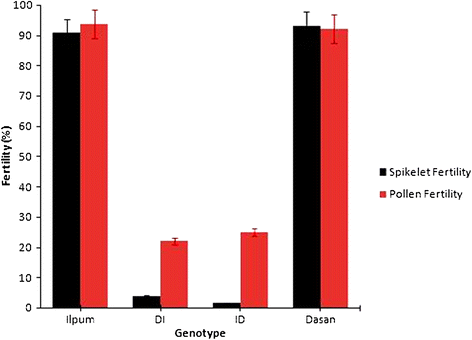

Supplement: Supplementary file 5 — Authors’ original file for figure 1 [file 12284_2014_3_MOESM5_ESM.gif]

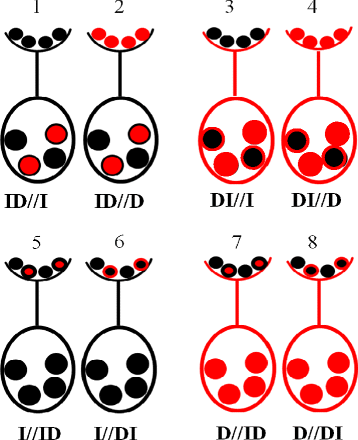

Supplement: Supplementary file 6 — Authors’ original file for figure 2 [file 12284_2014_3_MOESM6_ESM.gif]
